# Instrumentation of hypoplastic pedicles with patient-specific guides

**DOI:** 10.1007/s43390-024-00852-9

**Published:** 2024-04-01

**Authors:** Mazda Farshad, Christoph Zindel, Nico Akhavan Safa, José Miguel Spirig, Elin Winkler

**Affiliations:** Department of Spine Surgery, Balgrist University Hospital Zürich, University of Zürich, Forchstrasse 340, 8008 Zurich, CH Switzerland

**Keywords:** Hypoplastic pedicles, Patient-specific guides, Pedicle screws, Posterior spinal instrumentation, Complications

## Abstract

**Purpose:**

Hypoplastic pedicles of the thoracolumbar spine (<5 mm diameter) are often found in syndromic deformities of the spine and pose a challenge in pedicle screw instrumentation. 3D-printed patient-specific guides might help overcome anatomical difficulties when instrumenting pedicles with screws, thereby reducing the necessity for less effective fixation methods such as hooks or sublaminar wires. In this study, the surgical feasibility and clinical outcome of patients with hypoplastic pedicles following pedicle screw instrumentation with 3D-printed patient-specific guides were assessed.

**Methods:**

Hypoplastic pedicles were identified on preoperative computed tomography (CT) scans in six patients undergoing posterior spinal fusion surgery between 2017 and 2020. Based on these preoperative CT scans, patient-specific guides were produced to help with screw instrumentation of these thin pedicles. Postoperatively, pedicle-screw-related complications or revisions were analyzed.

**Results:**

93/105 (88.6%) pedicle screws placed with patient-specific guides were instrumented. 62/93 (66.7%) of these instrumented pedicles were defined as hypoplastic with a mean width of 3.07 mm (SD  ±0.98 mm, 95% CI [2.82–3.32]). Overall, 6 complications in the 62 hypoplastic pedicles (9.7%) were observed and included intraoperatively managed 4 cerebrospinal fluid leaks, 1 pneumothorax and 1 delayed revision due to 2 lumbar screws (2/62, 3.3%) impinging the L3 nerve root causing a painful radiculopathy. The mean follow-up time was 26.7 (SD  ±11.7) months. Complications were only noted when the pedicle-width-to-screw-diameter ratio measured less than 0.62.

**Conclusion:**

Patient-specific 3D-printed guides can aid in challenging instrumentation of hypoplastic pedicles in the thoracolumbar spine, especially if the pedicle-width-to-screw-diameter ratio is greater than 0.62.

## Introduction

Posterior spinal instrumentation and fusion with pedicle screw fixation is widely used for a variety of conditions [1, 2]. Nevertheless, different factors such as anatomical variations, as seen in hypoplastic pedicles, may increase the risk of intraoperative complications and pose a challenge during instrumentation [2–4]. Hypoplastic pedicles, here defined as pedicles measuring less than 5 mm at their thickest diameter in the axial view, are often found in syndromic deformities of the spine such as in neuromuscular disorders as well as connective tissue disorders including Loeys–Dietz syndrome or Marfan syndrome. However, they can also occur sporadically [5–8]. Hypoplasia and aplasia, seen in lumbar pedicles, are thought to represent an aborted attempt at the formation of a vertical cleft in the vertebral arch [9] and are frequently seen at the apical segments of scoliotic curves [10]. Other developmental abnormalities of pedicles include persistent neurocentral synchondrosis, cleft pedicles as well as complete or partial agenesis of the pedicle and neural arch [11].

The diameter of commercial pedicle screws used for instrumentation of the thoracolumbar region usually measures 5 mm in adults and 4 mm in children. Morphological studies have shown mean thoracolumbar pedicle widths usually to be greater than 5 mm [12, 13], with one study, however, showing T4–T8 diameters between 4.5 and 4.8 mm in a Japanese population [14]. Due to anatomical difficulties in hypoplastic pedicles, instrumentation of these pedicles is, therefore, often omitted to avoid neurovascular or pulmonary complications due to pedicle screws perforating the pedicle wall [15]. In addition, hypoplastic pedicles are more frequently sclerotic and the pedicle canal is, therefore, often occluded. In such cases, other fixation methods such as hooks, laminar polyester bands or sublaminar wires are used in place of pedicle screws [1, 16–18]. Pedicle screws, however, seem to be advantageous in achieving superior curve correction and curve maintenance, illustrating their importance in posterior spinal instrumentation [1, 18].

Patient-specific template-guided instrumentation has shown high accuracies when compared with other pedicle screw navigation techniques, and shorter surgery times have been noted. In addition to this, fewer perioperative complications have been observed [19–25]. Screw instrumentation with patient-specific guides in hypoplastic pedicles could, therefore, possibly aid screw insertion while achieving an acceptable rate of perforation-related complications. To our knowledge, surgical feasibility and clinical outcomes of patients with hypoplastic pedicles undergoing template-guided pedicle screw instrumentation have not been reported in the literature so far. Therefore, the aim of this case series was to demonstrate our approach, enabling pedicle screw instrumentation in spinal fusion surgeries of the thoracic and lumbar spine in patients with hypoplastic pedicles, which sometimes measured less than the screw diameter itself.

## Materials and methods

A total of six patients with hypoplastic pedicles undergoing posterior spinal instrumentation were identified at our institution from 2017 to 2020, resulting in a total of 105 instrumented pedicles, of which 93 were placed with patient-specific guides. In 62/93 (66.7%) pedicles, ranging from thoracic to lumbar spine segments, the pedicle width measured less than 5 mm on CT scans (Siemens SOMATOM Definition AS, Siemens Healthcare, Erlangen, Germany, slice thickness: 0.64 mm). The remaining 12 pedicles were instrumented with a free-hand technique under fluoroscopic guidance. Demographic and clinical data were obtained from the medical records and operation notes. Due to the already thin pedicles of the cervical spine as well as the separate instrumentation system used with smaller pedicle screw diameters (3–4.5 mm diameter), cervical spine segments were excluded from the analyses. Thus, this case series will focus only on the feasibility of patient-specific guides in hypoplastic pedicles of the thoracolumbar spine.

### Preoperative planning

**Fig. 1:**
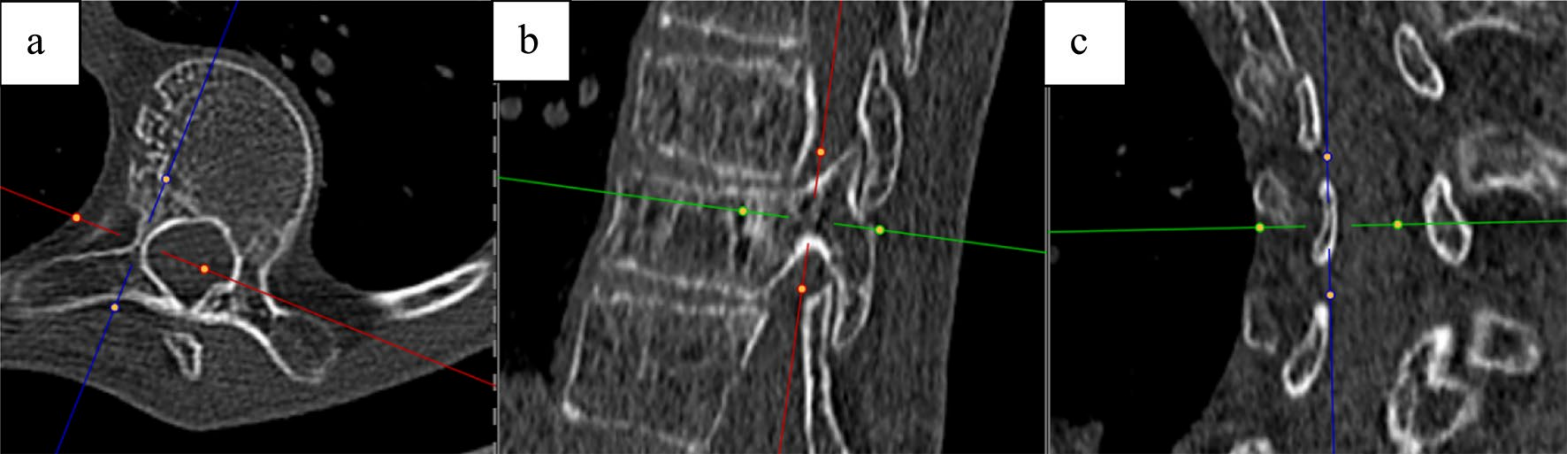
The axis chosen to measure the pedicle width on the axial view (**a**). A line parallel to the vertebral endplate in the sagittal plane (**b**) and in the axis of the pedicle on the coronal plane (**c**) was chosen. Afterward, in the axial view, a line in the middle of the pedicle was drawn and a perpendicular line chosen to measure the narrowest portion of the pedicle

**Fig. 2:**
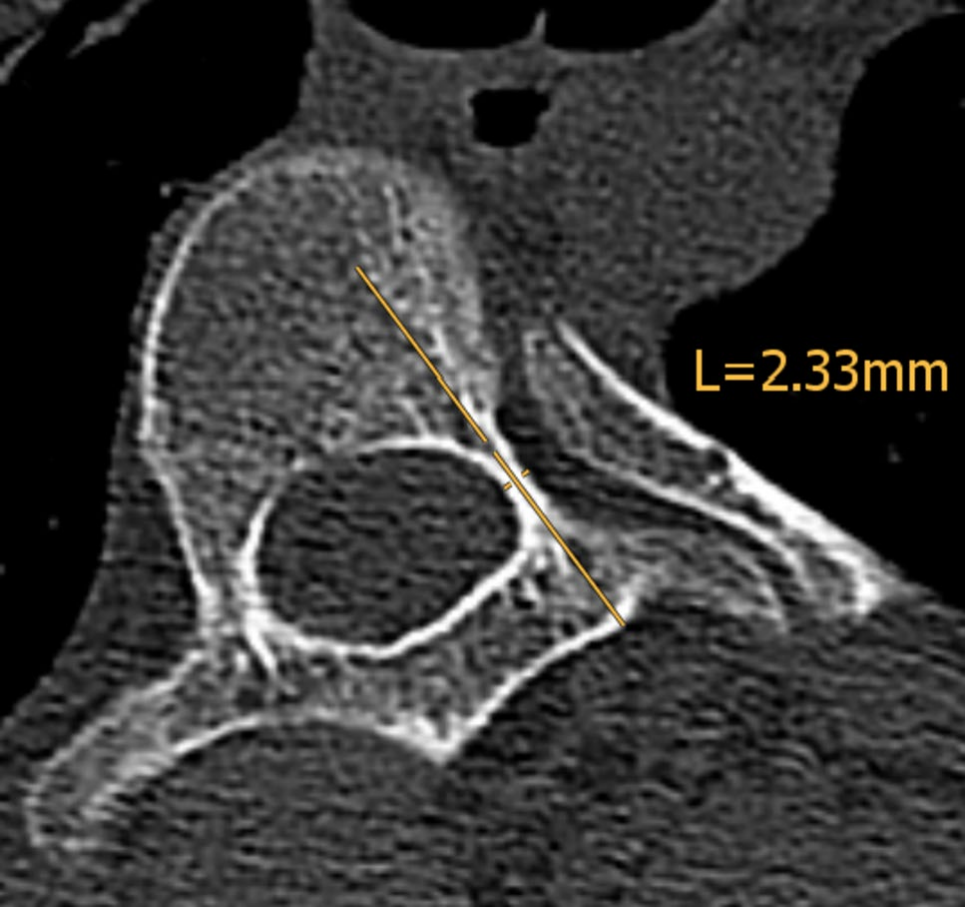
A line drawn in the middle of the pedicle representing the longitudinal axis of the pedicle. At the narrowest site, the diameter of the pedicle was measured from cortex to cortex

All patients were evaluated with neurological exams, bending radiographs, magnetic resonance imaging (MRI), and computed tomography (CT) scans (0.64 mm slice, Siemens SOMATOM Definition AS, Siemens Healthcare, Erlangen, Germany) preoperatively. Coronal, sagittal, and axial images were reformatted with a slice thickness of 3.0 mm and were sent to the PACS server. The criteria for measurements were similar to those used in the studies of Cadha et al. and Morita et al. [12, 14]. Linear measurements were taken from the CT images using dividers. The axial view was used to measure the pedicle width using a plane parallel to the vertebral endplates in the sagittal view and a line falling in the axis of the pedicles in the coronal view (Fig. 1). A longitudinal axis of the pedicle was placed by best visual fit through the middle of the pedicle on the axial view. At the narrowest portion, a line perpendicular to the pedicle longitudinal axis from cortex to cortex was placed and this value was used as a measure of the pedicle width (Figs. 1 and 2).

**Fig. 3:**
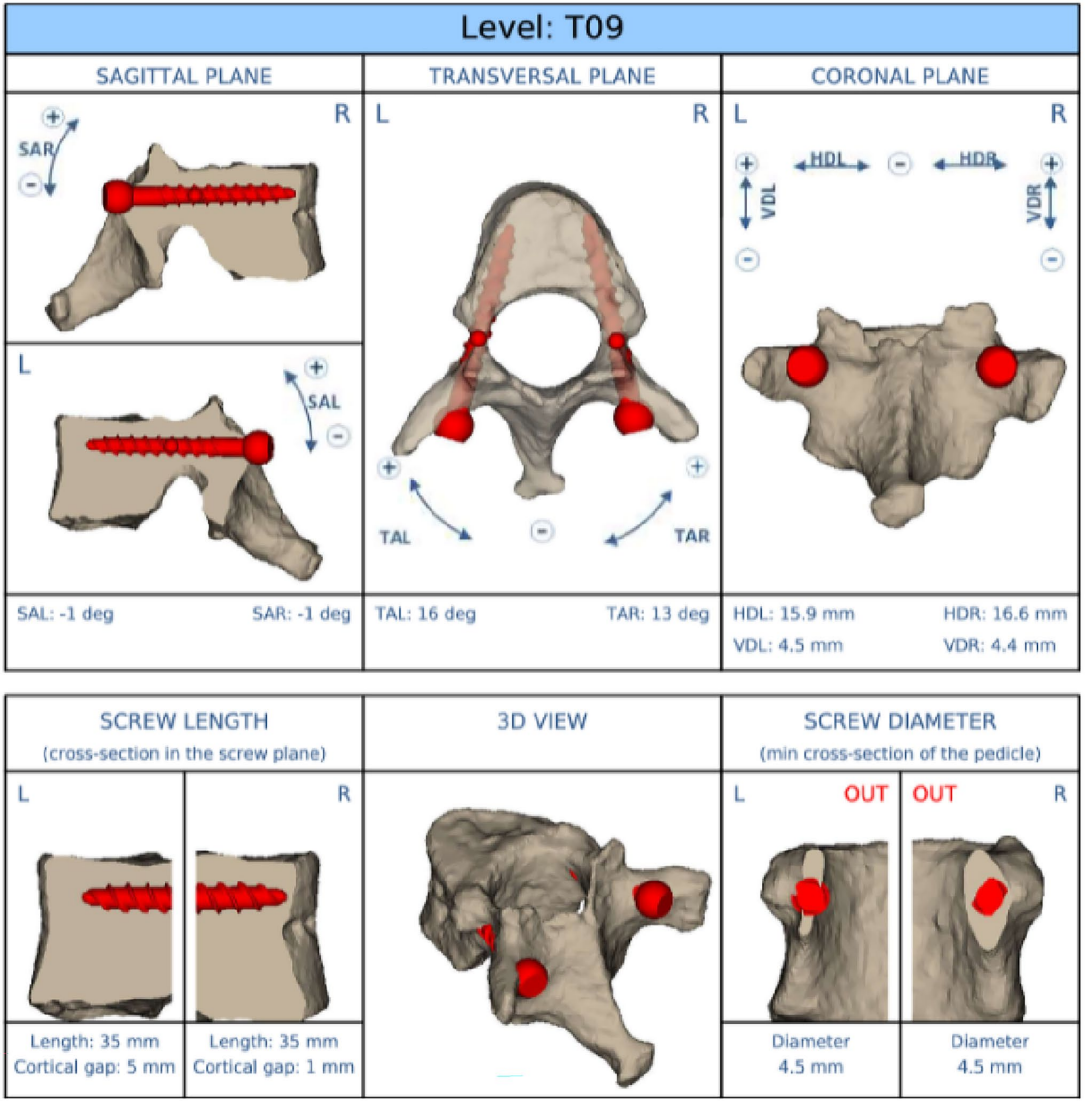
The plan of the chosen screw dimensions and trajectories. Notice the narrow, hypoplastic pedicles and the perforation of the medial and lateral wall even when using the smallest pedicle screws

**Fig. 4:**
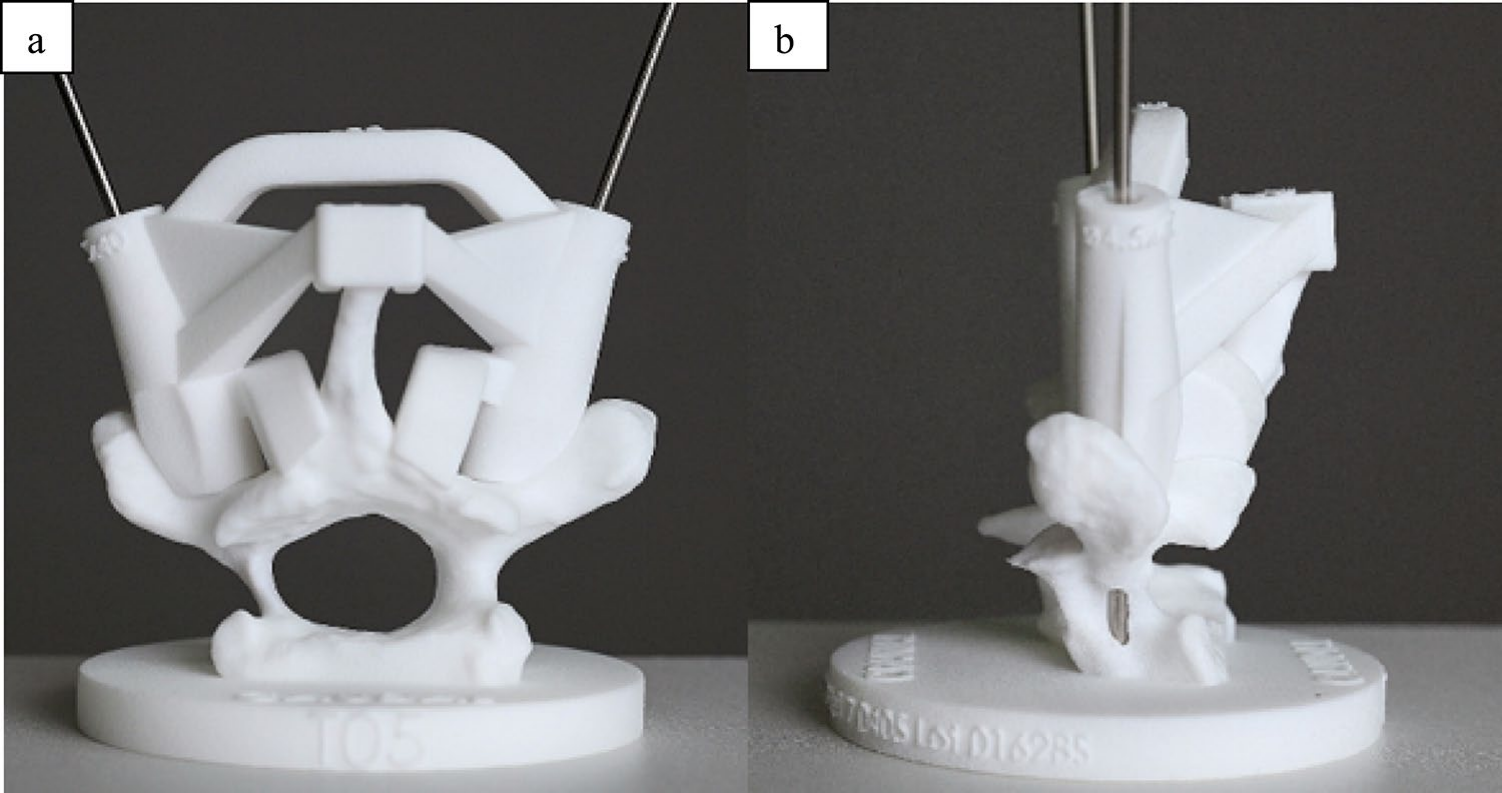
The 3D-printed, patient-specific drill guide on the vertebral model. Note the thin, dysplastic pedicle, and the lateral pedicle wall perforation by the k-wire due to the small diameter of the pedicle

Screw trajectories and dimensions were planned with the MySpine software (MySpine, Medacta SA International, Switzerland) based on preoperative CT scans (Fig. 3). These preoperative plans were later used to produce the patient‑specific 3D‑printed drill guides (MySpine, Medacta SA international, Switzerland) used for instrumentation of the pedicles (Fig. 4), a method that has been documented and validated in previous studies [22–26].

All CT scans were evaluated by two independent observers to calculate the inter-observer reliability. This was evaluated using a single-measure intraclass correlation coefficient (ICC) with a two-way random-effects model for absolute agreement. Descriptive statistics used frequencies and percentages to present the data. All the statistical analyses were performed using Excel Microsoft 365 and SPSS version 23 software (SPSS Inc., Chicago, Illinois).

### Surgical intervention

All procedures were performed by the same surgical team using a standard midline posterior surgical approach. To allow the guide to be properly placed on the vertebral body (transverse process, lamina and spinous process), the paraspinal muscle and any intervening soft-tissue was removed from the osseous structures. After correct placement of the guide on the bony landmarks, 2.7 mm burr holes were drilled through the guides with a COLIBRI II drill machine (DePuy Synthes, Switzerland). K-wires were then placed in the burr holes to guide the taping and later the instrumentation with canulated screws (Medacta, MUST System).

### Postoperative evaluation

All patients were followed-up clinically and with conventional radiographs, with a mean follow-up time of 26.7 months (SD ±11.7). The pedicle widths measured on preoperative CT scans as described above were compared to the diameter of the inserted pedicle screws to calculate the pedicle-width-to-screw-diameter ratio. The data were then analyzed for any intraoperative or postoperative complications and the highest pedicle-width-to-screw-diameter ratio where complications still occurred was determined. Furthermore, the need for revision surgery was analyzed.

## Results

In total, 6 patients (3 syndromic scoliosis, 1 idiopathic scoliosis, 2 tumors) underwent posterior spinal instrumentation resulting in 105 instrumented pedicles, of which 62 were hypoplastic, meaning the pedicle width measured less than 5 mm at its narrowest portion. These 62 hypoplastic pedicles were instrumented with patient-specific guides. Further information regarding patient demographics is listed in Tables 1 and 2. The thinnest pedicles were observed most often at L1, in average measuring 2.05 mm (SD ±0.07, 95% CI [1.99–2.11]), while the minimal width of 0.96 mm was noticed on a right-sided pedicle of T4 (Table 3). Thirteen pedicles measured 4–5 mm, followed by seven, twenty-two, and ten pedicles measuring 3–4 mm, 2–3 mm, and below 2 mm, respectively. T4–T6 were the levels which were most frequently affected by hypoplastic pedicles and instrumented with patient-specific guides (29/62, 47%). The majority of the instrumented hypoplastic pedicles (50/62, 81%) were located in the thoracic spine, while the remaining belonged to the lumbar spine (12/62, 19%). The inter-observer reliability was in perfect agreement for the measurement of the pedicle width (0.989).

**Table 1:** Patient demographics

Patient Nr	Age at operation	Sex	Indication	Intervention date	Spinal pathology
1	16	M	Syndromic scoliosis	11/2019	Thoracolumbar kyphoscoliosis caused by an unidentified dysmorphic syndrome: • Thoracolumbar scoliosis (apex: T9, Cobb angle: 64°) • Kyphosis (apex: T9, sagittal Cobb angle: 80°)
2	16	M	Idiopathic scoliosis	06/2019	Double thoracic idiopathic adolescent scoliosis: • Proximal thoracic, right convex T2–6 (apex: T4, Cobb angle: 48°) • Main thoracic, left convex T7–12 (apex: T10, Cobb angle: 55°) • Lenke type 2, lumbar spine modifier A
3	14	F	Syndromic scoliosis	05/2019	Residual right-sided convex thoracic scoliosis in a patient with Marfan syndrome and magnetic growing rod in 2013: • Apex: T11–12, Cobb angle: 64°
4	16	F	Syndromic scoliosis	10/2018	Thoracic hyperkyphosis after thoracolumbar anterior scoliosis correction T12–L3 in 2015 in patient with Loeys–Dietz syndrome (LDS) • Apex: T5, sagittal Cobb angle: 62°
5	64	F	Tumor	12/2017	Residual cervical myelopathy C5/C6 with tetraparesis below C4 after corpectomy C5–C7 and anterior plate fixation C4–T1 in 11/2017 due to breast cancer metastases
6	78	F	Tumor	08/2020	Complete pathologic burst fracture of T2 and T3 with sintering in a patient with metastases of an ovarian endometrial adenocarcinoma with therapy resistant pain

**Table 2:** Interventions, complications, and postoperative neurologic examination

Patient Nr	Procedure	Levels instrumented with guides	Intraoperative complications	Preoperative neurological exam	Postoperative neurological exam
1	Posterior instrumentation and fusion T3–4	T3–L3	None	Normal	Normal
2	Posterior instrumentation and fusion T2–L2	T2–T7	None	Normal	Normal
3	Posterior instrumentation and fusion T1–L4 in fully grown patient	T1–L4	L3 both sides: cerebral spinal liquor leak after screw hole placement. Leak ceased by screw insertion	Normal	5 months postoperatively left-sided painful L3 radiculopathy
4	Posterior instrumentation and fusion T3–L6	T3–L6	T4 right side: pneumothorax—fibrin sealant patch and screw insertion T6 both sides: cerebral spinal liquor leak—fibrin sealant patch	Normal	Normal
5	Posterior instrumentation and posterolateral fusion with allograft C3–T5	C3–C4 T1–T2 T5–T6	None	AISA D below C4	AISA D below C4
6	Posterior instrumentation and posterior fusion C6–T6	C6–T1 T4–T6	None	Hyposensibility in all fingers of both hands	None

**Table 3:** Number and types of pedicles instrumented, screw and pedicle dimensions, and complications noted

Pedicle	Total number of interventions on specific level in case series group	Minimal measured pedicle width within case series group	Use of patient-specific guides	Complications	Average pedicle width	Minimal used screw diameter	Standard deviation of pedicle width
T1R	3	4.42	3	0	6.05	4.5	0.95
T1L	3	5.94	3	0	7.14	4.5	1.12
T2R	3	3.58	3	0	4.05	4.0	0.79
T2L	3	3.99	3	0	4.54	4.0	0.90
T3R	3	1.89	3	0	3.14	4.0	1.54
T3L	3	2.22	3	0	3.97	4.0	1.63
T4R	4	0.96	4	1	4.01	4.0	1.78
T4L	4	1.24	4	0	2.90	4.0	0.91
T5R	6	1.54	6	0	3.95	4.5	1.75
T5L	4	2.00	4	0	3.26	4.5	0.65
T6R	6	1.79	6	1	4.46	4.5	1.45
T6L	5	2.40	5	1	3.87	4.5	0.88
T7R	2	2.52	2	0	4.57	4.5	1.85
T7L	2	2.75	2	0	4.26	4.5	1.30
T8R	3	2.71	2	0	3.50	4.5	0.90
T8L	2	2.36	2	0	4.09	4.5	0.66
T9R	1	2.32	1	0	3.37	4.5	1.14
T9L	2	1.25	1	0	3.68	4.5	1.71
T10R	4	2.56	3	0	4.52	4.5	1.58
T10L	2	2.29	2	0	4.39	4.5	0.67
T11R	3	3.17	3	0	4.78	4.5	0.76
T11L	4	2.53	3	0	5.15	4.5	2.23
T12R	2	1.61	1	0	3.97	4.5	2.35
T12L	2	1.78	1	0	3.73	4.5	1.68
L1R	3	1.71	2	0	2.55	4.5	0.64
L1L	3	1.23	2	0	2.05	4.5	0.07
L2R	3	0.99	2	0	2.79	4.5	0.30
L2L	4	1.26	3	1	2.52	4.5	0.82
L3R	3	2.73	3	1	3.40	4.5	0.57
L3L	3	1.94	3	1	3.36	4.5	1.60
L4R	3	1.54	2	0	3.28	4.5	2.02
L4L	3	1.42	2	0	3.31	4.5	2.61
L5R	1	5.68	1	0	6.70	4.5	–
L5L	1	6.32	1	0	5.60	4.5	–
L6R	1	5.6	1	0	5.6	5.0	–
L6L	1	6.7	1	0	6.7	5.0	–

While 4.5 mm screws were used most often (52/62, 84%), the maximal and minimal screw diameter measured 5 mm (8/62, 13%) and 4 mm (2/62, 3%), respectively. The average ratio of pedicle-width-to-screw-diameter of all hypoplastic pedicles was 0.67 (SD ±0.21, 95% CI [0.62–0.73]). The minimal encountered ratio was 0.32, where a 4.5 mm screw was inserted into a 1.41 mm thick pedicle (L4) of a patient with syndromic scoliosis.

**Fig. 5:**
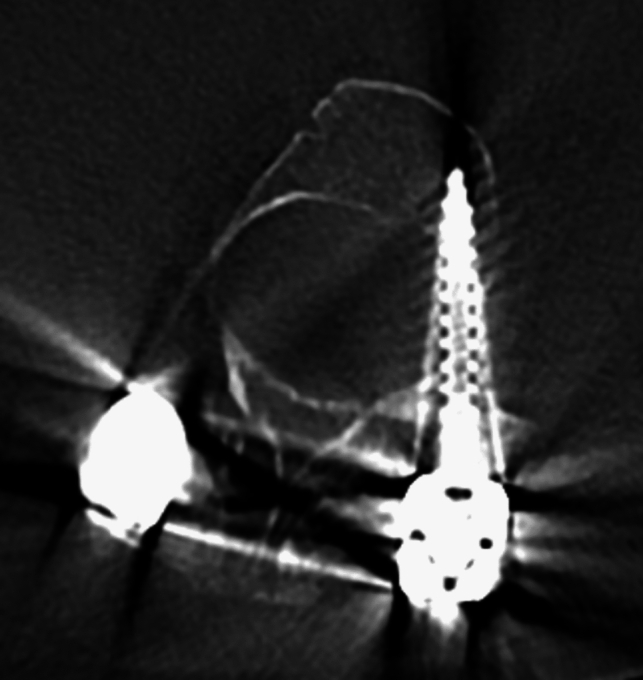
A recessal position of the pedicle screw causing impingement of the left L3 nerve root

Overall, six (three thoracic, three lumbar) complications (6/62, 9.7%) in two out of the six patients occurred during instrumentation of hypoplastic pedicles with a mean width of 1.91 mm (SD ±0.75, 95% CI [1.31–2.51]). Complications included four cerebrospinal fluid leaks and one pneumothorax (Table 4). All of these were noted intraoperatively after removing the drill out of the burr hole and observing either cerebrospinal fluid or air bubbles exiting the burr hole. All four cerebrospinal fluid leaks resolved after inserting TachoSil® (Human Thrombin, Human Fibrinogen absorbable collagen fibrin sealant patch) or TISSEEL® (human fibrinogen and thrombin based frozen sealant) and a screw into the burr hole. The observed pneumothorax was also managed by inserting TachoSil into the burr hole followed by a screw into the pedicle. After surgery, the pneumothorax was confirmed with a chest X-ray and a chest tube was inserted. Removal of the chest tube followed 2 days later after confirming complete resolution of the pneumothorax on chest X-ray. One patient (1/6, 16.7%) needed revision surgery 9 months after the initial operation for an intermittent, painful left-sided L3 radiculopathy due to a nerve root impingement (Fig. 5) caused by a misplaced L2 and L3 screw (2/62, 3.2%). Complete resolution was achieved after removal of the affected screws.

**Table 4:** Complications

Patient	Segment	Intraoperative complications	Therapy	Later reoperations	Ratio pedicle-width-to-screw-diameter
3	L2 left L3 left	Cerebrospinal fluid leak	Screw insertion	Removal of left L2 and L3 screw due to painful L3 radiculopathy	0.28
					0.43
3	L3 right	Cerebrospinal fluid leak	Screw insertion	None	0.61
4	T4 right	Pneumothorax	Sealant patch, Chest tube	None	0.21
4	T6 right	Cerebrospinal fluid leak	Sealant patch	None	0.40
4	T6 left	Cerebrospinal fluid leak	Sealant patch	None	0.62

**Fig. 6:**
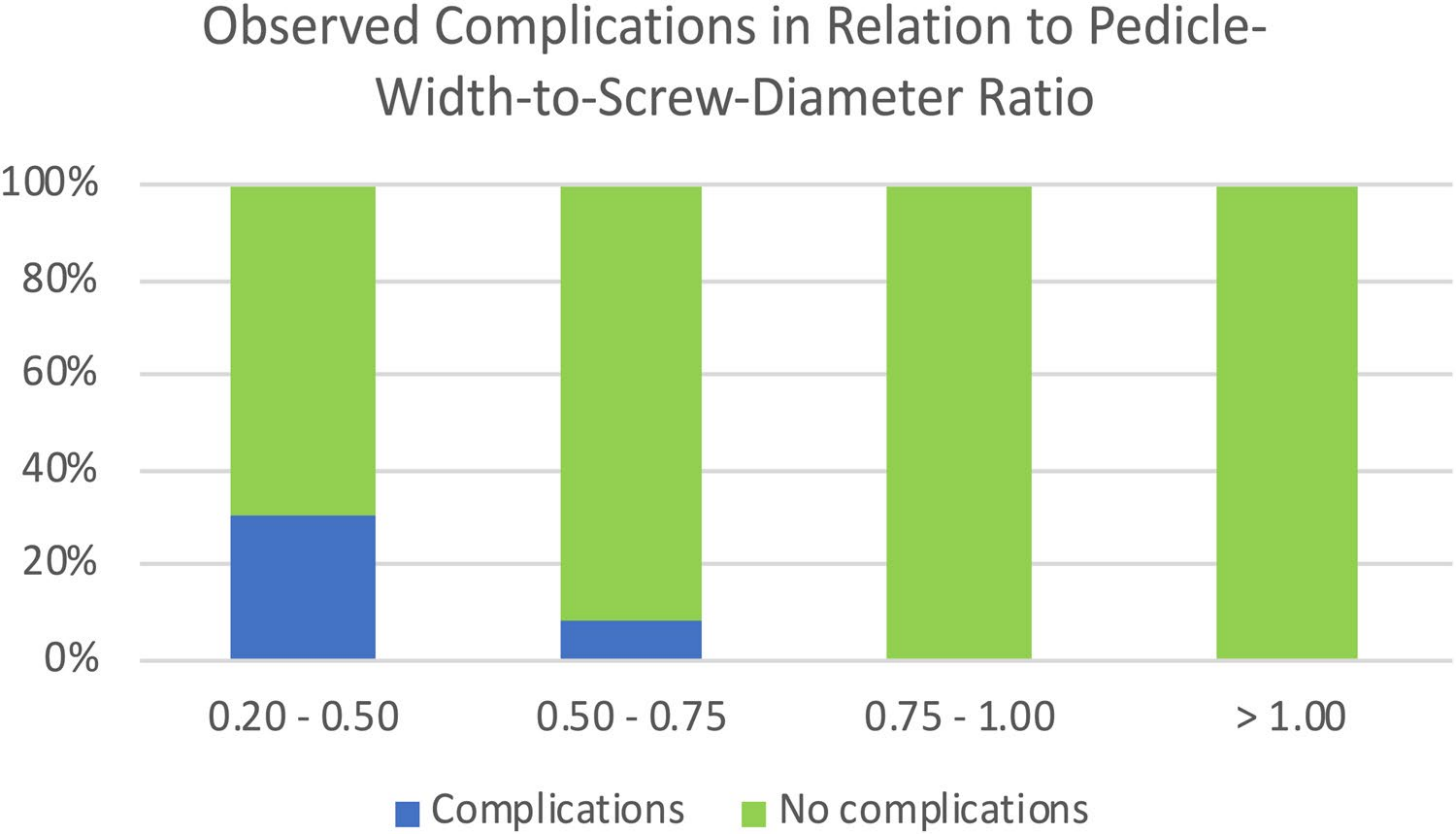
The observed complications (%) relative to the pedicle-width-to-screw diameter ratio

**Fig. 7:**
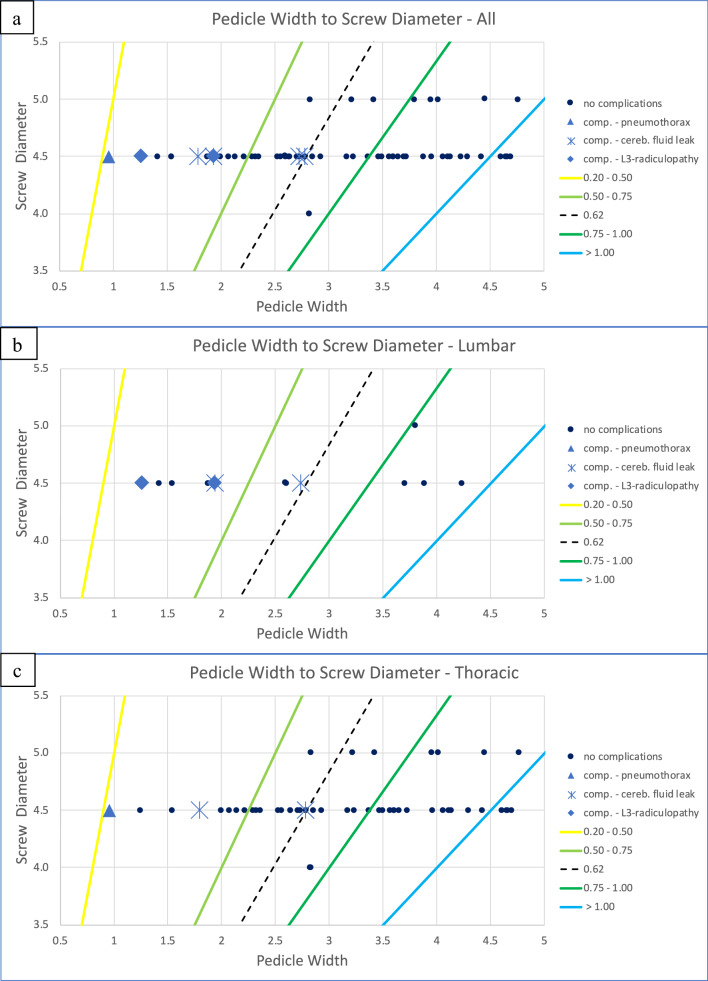
The cut-off value of the pedicle-width-to-screw-diameter ratio where complications occurred for **a** all, **b** lumbar, and **c** thoracic pedicles. No complications below a ratio of 0.62 were observed

As shown in Fig. 6, complications occurred in 30.8% (4/13) and 8.3% (2/24) of the cases when the pedicle-width-to-screw-diameter ratio measured between 0.2–0.5 and 0.51–0.75, respectively. Complications were only noted when the ratio measured less than 0.62, which was valid for the thoracic as well as the lumbar spine segments (Fig. 7). The mean pedicle-width-to-screw-diameter ratio associated with complications overall was 0.45 (SD ±0.17).

## Discussion

The current study has demonstrated that hypoplastic pedicle screw instrumentation of the thoracolumbar spine with patient-specific guides is feasible when considering the technical difficulties. Hypoplastic or aplastic pedicles are often found in syndromic deformities, but can also occur sporadically [5, 6, 8, 9, 11]. Instrumentation of narrow pedicles is prone to complications and studies have shown that even when placed by an experienced spine surgeon, only 73% of the screws in neuromuscular scoliosis were placed accurately and 7% were outside the defined safe zone [27]. Furthermore, Uprenda et al. analyzed the screw positions of thoracic pedicles in scoliotic and non-scoliotic patients and noticed 90% and 87% showing an acceptable placement, respectively [28]. However, of misplaced screws, less than 1% seem to cause neurologic symptoms [29]. Other complications of pedicle screw instrumentation include pedicle fractures, screw breakage, and loosening ranging from 0.5 to 1% [29, 30] as well as vascular injury, pleural tear, and increased radiation exposure specifically during screw placement of thoracic pedicles [31]. The results of this study showed a complication rate of 9.7% following instrumentation of hypoplastic pedicles with patient-specific guides due to misplaced screws, all of which were resoluble after appropriate management. Furthermore, only one patient required revision surgery for two misplaced (2/62, 3.2%) screws. All these complications occurred only after falling below a pedicle-width-to-screw-diameter of 0.62.

In some cases, instrumentation of hypoplastic pedicles might have been impossible or too dangerous without the use of any type of navigation. As patient-specific guides are regarded as an accurate method of navigation [23, 32, 33], we see them as an optimal tool when instrumentation of such pedicles is planned. Further advantages of patient-specific guides include shorter surgical time, less radiation exposure, and decreased blood loss than the free-hand technique [34]. Shortcomings are the need of a more meticulous dissection of the soft-tissue from the bone for proper guide placement and the longer production time of the guides, which might be problematic in emergency cases [21, 35].

Other types of spinal navigation techniques include robotic-assisted and computer-assisted techniques, intraoperative image guidance or augmented reality [24, 27, 36–40]. Depending on the imaging used, the level of instrumentation, the definition of a pedicle breach, and the patient population, results regarding the misplacement of pedicle screws can vary. However, regarding robotic-assisted guidance of pedicle screw positioning, Macke et al. reported a breach greater than 2 mm in 7.2% in adolescent idiopathic scoliosis surgery [36]. In computer-assisted navigation, Amiot et al. noticed in postoperative MRI scans, 5.4% (16/294) of pedicle screws in neuromuscular scoliosis and 1.2% in unspecified spinal deformity to be misplaced [41]. Others, however, reported a perforation rate of 14% [37]. Newer techniques such as augmented reality have yielded promising results in cadaver experiments with 97.5% of screws being placed in the defined safe zone [38, 42]. All these studies aimed to analyze the precision of screw placement with radiographic images, rather than observing the clinical outcome and as a consequent, the possible need for revision surgery. In addition, a direct comparison between these different techniques is difficult, as the population groups in the abovementioned studies are very heterogenous and the definition as well as the extent of hypoplastic pedicles was not further quantified in these studies. All complications in our study occurred in patients with an underlying syndromic pathology. These syndromic pathologies are frequently associated with a distorted anatomy of the spine and smaller, dysplastic pedicle are often noted. This might explain the higher rate of misplaced screws causing complications (9.7%) in our study.

Regardless of the anatomy of the patient, when using patient-specific drill guides for the instrumentation of pedicles, to reduce the risk of misplacing screws, the surgeon should correctly plan the screws with adequate preoperative CT scans, perform a meticulous soft-tissue dissection while preserving the bony surface, firmly press the guide in the correct position on the bone and/or use a burr to decorticate the starting point to reduce movement between the guide and the bone while drilling [22, 24].

When pedicle screw instrumentation is not amenable, there are alternative, less rigid fixation techniques such as sublaminar wires, hooks, and laminar polyester bands [10]. However, these techniques pose some risk of harming the spinal canal [43, 44]. Spinal cord injuries after the use of sublaminar polyester bands have been reported in up to 10%, questioning its safety [45].

While some studies show similar results between sublaminar fixation and hook instrumentation [10, 44, 46, 47], other studies suggest an increased rate of correction when using pedicle screws [1, 17, 29, 48]. Kim et al. compared instrumentation using hooks with pedicle screws and not only showed a significantly greater curve correction, but also better maintenance without neurologic problems [1]. Similar results were shown in the study of Watanabe et al., where correction of curves greater than 100° in AIS using hooks, pedicle screws, and sublaminar wires was analyzed [18]. Cheng et al. compared pedicle screws and sublaminar wires in adolescent idiopathic scoliosis and noticed similar results regarding curve correction; however, less blood loss was observed when using pedicle screws [49].

Some limitations of this study include the small sample size, the lack of a control group as well as the heterogenic patient group consisting of different age groups, gender, etiology of spinal pathology, and surgical indications. Yet, in total 105 pedicles, of which 62 were still defined as hypoplastic and instrumented with patient-specific guides, compensated for the rather small population group (6 patients). Furthermore, we first wanted to evaluate the feasibility of patient-specific instrumentation of hypoplastic pedicles in a small group of patients. The prevalence of hypoplastic pedicles in the general population is low, contributing to the smaller patient group. In addition, we did not routinely perform a postoperative CT scan to reduce unnecessary radiation exposure in the young patient. Further imaging was only ordered in case of clinical symptoms. Therefore, we did not analyze screw placement radiographically in all instrumented pedicles; however, that was also not the aim of this study.

## Conclusion

Instrumentation of hypoplastic pedicles with patient-specific guides seems to be feasible down to a pedicle-width-to-screw-diameter ratio of 0.62. In the here presented study, instrumentation with patient-specific guides has proven to be a valuable surgical technique, utilizing the advantages of pedicle screws while minimizing potential complications.

## Data Availability

Data supporting this study are included within the article and/or supporting materials. If more data is necessary, it can be available on reasonable request.
